# Development of Double Hydrophilic Block Copolymer/Porphyrin Polyion Complex Micelles towards Photofunctional Nanoparticles

**DOI:** 10.3390/polym14235186

**Published:** 2022-11-29

**Authors:** Maria Karayianni, Dimitra Koufi, Stergios Pispas

**Affiliations:** Theoretical and Physical Chemistry Institute, National Hellenic Research Foundation, 48 Vassileos Constantinou Avenue, 116 35 Athens, Greece

**Keywords:** double hydrophilic block copolymers, porphyrins, polyion complex micelles, photosensitizers

## Abstract

The electrostatic complexation between double hydrophilic block copolymers (DHBCs) and a model porphyrin was explored as a means for the development of polyion complex micelles (PICs) that can be utilized as photosensitive porphyrin-loaded nanoparticles. Specifically, we employed a poly(2-(dimethylamino) ethyl methacrylate)-*b*-poly[(oligo ethylene glycol) methyl ether methacrylate] (PDMAEMA-*b*-POEGMA) diblock copolymer, along with its quaternized polyelectrolyte copolymer counterpart (QPDMAEMA-*b*-POEGMA) and 5,10,15,20-tetraphenyl-21H,23H-porphine-p,p′,p″,p′′′-tetrasulfonic acid tetrasodium hydrate (TPPS) porphyrin. The (Q)PDMAEMA blocks enable electrostatic binding with TPPS, thus forming the micellar core, while the POEGMA blocks act as the corona of the micelles and impart solubility, biocompatibility, and stealth properties to the formed nanoparticles. Different mixing charge ratios were examined aiming to produce stable nanocarriers. The mass, size, size distribution and effective charge of the resulting nanoparticles, as well as their response to changes in their environment (i.e., pH and temperature) were investigated by dynamic and electrophoretic light scattering (DLS and ELS). Moreover, the photophysical properties of the complexed porphyrin along with further structural insight were obtained through UV-vis (200-800 nm) and fluorescence spectroscopy measurements.

## 1. Introduction

Successful cancer treatment is one of the most sought-after goals of modern-day medicine, since cancer remains a leading cause of death worldwide, accounting for nearly 10 million deaths in 2020 according to the World Health Organization [1]. Among the various proposed therapeutic strategies, photodynamic therapy (PDT) holds significant promise owing to its efficiency, site-specificity, and noninvasive characteristics. It is being used not only for the treatment of tumors related to various types of cancers (e.g., skin, esophageal, lung) but also a number of other diseases and medical conditions, such as atherosclerosis, rheumatoid arthritis, macular degeneration, psoriasis, and acne [2]. The working principle of PDT is based on the administration of photosensitizers that accumulate in pathological tissue and are subsequently exposed to a light source with appropriate wavelength so as to generate the production of reactive oxygen species (ROS), which in turn cause cell death [3,4,5,6]. Due to its selective action, it causes minimal damage to the surrounding healthy tissue, has no severe local or systemic side effects, is not painful, is well tolerated by patients, allows for outpatient use, and can even be applied in parallel with other therapeutic protocols [2,3]. All these advantages in combination with documented good therapeutic results render PDT a widespread contemporary method for the treatment of cancer and other infectious diseases.

Evidently, the role of the photosensitizer is of utmost importance for the effective application of PTD. Having entered the cell, the photoactive molecule is appropriately irradiated, leading to its excitation from the ground to the excited singlet state as a result of the photon absorption [3,4,5]. Next, the excited photosensitizers either relax back to the ground state via fluorescence photon emission or are transformed to the excited triplet state, whose energy under well-oxygenated conditions can be transferred to the surrounding tissue oxygen molecules, mainly producing singlet oxygen (^1^O_2_). These singlet oxygen particles are characterized by extremely strong oxidizing properties and can destroy tumor cells, either directly by inducing apoptotic and/or necrotic cell death or indirectly by provoking autophagy [3,4]. Over the years of PDT clinical practice, several characteristics have been established as prerequisites for ideal photosensitizers. These include high chemical purity, stability at room temperature, high photochemical reactivity, minimum dark cytotoxicity, preferential retention at the targeted tissue, facile solubility in bodily fluids, low cost, and wide availability, among others [2,3]. Of course, the most widely used photosensitizers consist of porphyrins (a class of heterocyclic macrocycle organic compounds) and their derivatives, since the historic discovery of hematoporphyrin and its photo-related medical properties in the beginning of the previous century [2].

Owing to their hydrophobic nature, porphyrins tend to self-assemble in aqueous media, forming aggregates of intriguing structures and unique features [7]. Nevertheless, aggregation reduces their photoactivity and thus therapeutic efficiency. In order to enhance porphyrin water solubility, as well as circulation lifetime and tumor specificity, and at the same time reduce aspecific tissue accumulation, the use of various types of polymeric nanocarriers has been proposed [4,5,6,8]. Among the plethora of available macromolecular architectures, the use of double hydrophilic block copolymers (DHBCs)—consisting of one charged and one hydrophilic block—as delivery vehicles is of particular interest due to their interesting self-assembly behavior [9,10,11,12]. This kind of copolymer is able to interact with oppositely charged bioactive species, forming polyion complex micelles (PICs) where the interacting charged species form the micellar core and the hydrophilic blocks the corona, thus providing solubility or even biocompatibility and stealth properties to the formed nanoparticles. This principle has been extensively exploited over the years as a means of developing porphyrin-loaded micelles for potential PDT applications. Starting with the work of Kataoka (a pioneer in PICs) and his coworkers on dendritic zinc porphyrins [13,14], followed by the extended studies of the Shi group on 5,10,15,20-tetrakis-(4-sulfonatophenyl)-porphyrin (TPPS) and its metal ions [15,16,17,18,19,20], as well as numerous other similar investigations involving not only DHBC [21,22,23,24,25,26] but also surfactants [27,28], polycations [29], nonionic triblock copolymers [30], or even polymeric membranes [31], one can only begin to fathom the importance of these systems considering their prospective therapeutic capability, and thus justify the ongoing considerable scientific interest they have attracted.

In this work, we report on the formation of photofunctional nanoparticles (owing to the intrinsic properties of the porphyrin) through the electrostatic complexation between the poly(2-(dimethylamino) ethyl methacrylate)-*b*-poly[(oligo ethylene glycol) methyl ether methacrylate] (PDMAEMA-*b*-POEGMA) double hydrophilic block copolymer or its quaternized strong polyelectrolyte counterpart (QPDMAEMA-*b*-POEGMA) and 5,10,15,20-tetraphenyl-21H,23H-porphine-p,p′,p″,p′′′-tetrasulfonic acid tetrasodium hydrate (TPPS) porphyrin. The resulting PIC micelles comprise a mixed polyelectrolyte–porphyrin (Q)PDMAEMA/TPPS core and a hydrophilic biocompatible POEGMA corona/shell. Their solution properties in regard to their mass, size, size distribution and effective charge at varying mixing ratios with increasing porphyrin content were thoroughly investigated by means of dynamic and electrophoretic light-scattering techniques. In parallel, UV-vis and fluorescence detailed spectroscopic studies were conducted to probe the photophysical characteristics of the complexed porphyrin, extract additional information regarding its morphology and aggregation state, and of course validate the photosensitivity of the produced nanoparticles. The pH-dependent charge density of the PDMAEMA polyelectrolyte and aggregation behavior of TPPS porphyrin necessitated the investigation to be performed at different pH values, namely, pH 7 and 3. Moreover, the effect of temperature on the overall properties of the already formed PICs was examined.

## 2. Materials and Methods

### 2.1. Materials

**Scheme 1:**
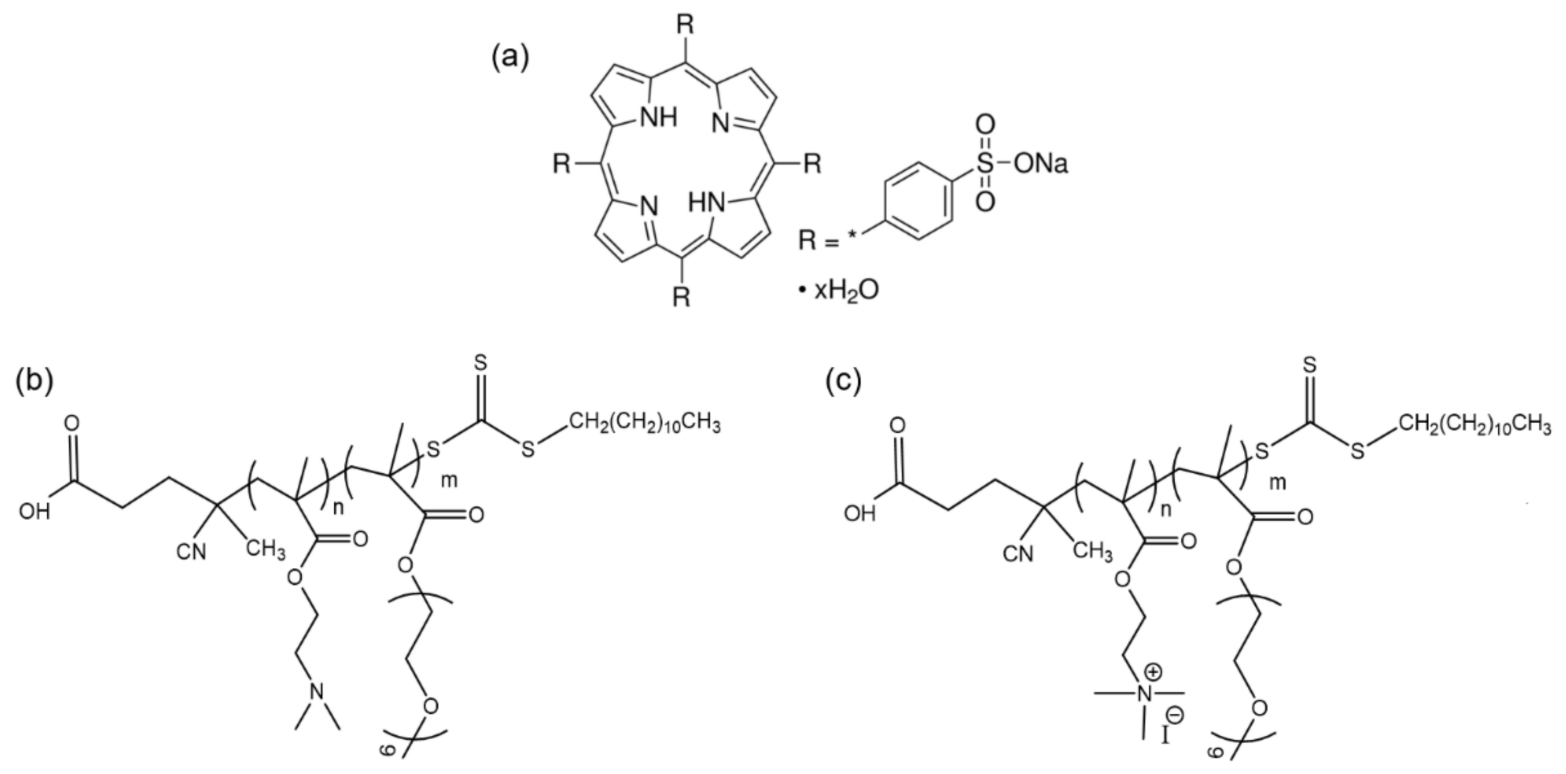
Molecular structures of (**a**) TPPS porphyrin, (**b**) PDMAEMA-*b*-POEGMA copolymer and (**c**) its quaternized counterpart QPDMAEMA-*b*-POEGMA.

The 5,10,15,20-tetraphenyl-21H,23H-porphine-p,p′,p″,p′′′-tetrasulfonic acid tetrasodium hydrate (TPPS) porphyrin (*M*_w_ = 1022.92 g/mol, anhydrous basis) (Scheme 1a) and all other reagents were purchased from Sigma-Aldrich (St. Louis, MO, USA) and used as received.

### 2.2. Block Copolymer Synthesis and Quaternization

The synthetic procedure of the PDMAEMA-*b*-POEGMA block copolymer by means of reversible addition fragmentation chain transfer (RAFT) polymerization, as well as the subsequent quaternization reaction, were performed in a similar manner to the one described in detail in a previous publication [32]. In brief, the synthetic route comprises two steps, starting with the initial polymerization of the DMAEMA monomer under appropriate conditions and the formation of the PDMAEMA homopolymer, which then acts as a macro-chain transfer agent (macro-CTA) for the polymerization of the OEGMA monomer (which bears 9 ethylene glycol units) and the formation of the POEGMA block. The molecular characteristics of the resulting copolymer as determined by SEC and ^1^H NMR are summarized in Table 1, while its molecular structure is shown in Scheme 1b. The quaternization of the PDMAEMA-*b*-POEGMA precursor was performed by appropriate reaction with methyl iodide (CH_3_I), thus transforming the tertiary amines of the PDMAEMA block to quaternary ammonium groups, as seen in Scheme 1c. The corresponding molecular characteristics are also presented in Table 1.

### 2.3. Preparation of the Polyion Complex Micelles (PICs)

Initially, stock solutions of the PDMAEMA-*b*-POEGMA copolymer or its quaternized counterpart QPDMAEMA-*b*-POEGMA at a concentration of 1 mg/mL and TPPS porphyrin at 0.53 mg/mL in water for injection (WFI) were prepared (magnetic stirring was usually employed in order to assist solubilization) and left overnight at ambient conditions to equilibrate. The pH of these solutions was around 6.5, henceforth called pH 7 in the following. For the formation of the complexes, appropriate aliquots, namely, 0.4, 1, 1.4, and 2 mL, of the porphyrin solution were added to 2 mL of the copolymer solution under stirring, mixing of the two constituent solutions was continued for 5 min, and at the final stage, dilution with WFI to a total volume of 10 mL was performed. In this way, the copolymer concentration is kept constant throughout the series of complex solutions, while porphyrin concentration increases thus changing the molar ratio of the two components. The resulting solutions were stable and no precipitation was observed. The stock solution concentration and mixing ratios were suitably chosen so as the molar ratio of the TPPS sulfonate groups to the corresponding tertiary amine groups of the DHBC or quaternary ammonium groups of its quaternized counterpart (QDHBC)—denoted as SO_3_^−^/NR_2_ or NR_3_^+^, where R stands for the methyl group—ranged from 20% to 100%. The characteristics of the complex solutions in regard to the final concentration of the components and charged groups ratio are given in Table 2. Exactly the same procedure was followed for the preparation of the corresponding complexes at pH 3, starting with copolymer and porphyrin stock solutions whose pH was adjusted to 3 by appropriate addition of 0.1 M HCl, leading again to stable complex solutions.

### 2.4. Methods

*Dynamic Light Scattering (DLS).* DLS measurements were performed on an ALV/CGS-3 compact goniometer system (ALVGmbH, Hessen, Germany) equipped with an ALV-5000/EPP multi-tau digital correlator, a He-Ne laser (*λ* = 632.8 nm), and an avalanche photodiode detector. All sample solutions were filtered through 0.45 μm hydrophilic PVDF syringe filters (ALWSCI Group, Hangzhou, China) before measurement in order to remove any dust particles or large aggregates. The samples were loaded into standard 1 cm width soda-lime glass dust-free cylindrical cells and measurements were performed at a series of angles in the range of 45–135°. A circulating water bath was used to set the temperature at 25 °C, or at the desired value (25–60 °C) in the case of temperature-dependent measurements.

The measured normalized time autocorrelation functions of the scattered light intensity *g*_2_ (*t*) were fitted with the aid of the CONTIN analysis, thus obtaining the distribution of relaxation times *τ*. Assuming that the observed fluctuations of the scattered intensity are caused by diffusive motions, the apparent diffusion coefficient *D*_app_ is related to the relaxation time *τ* as *D*_app_ = 1/*τq*^2^, where *q* is the scattering vector defined as $4\pi n_{0}\sin(\theta /2)/\lambda_{0}$ with *n*_0_, *θ* and *λ*_0_ the solvent refractive index, the scattering angle, and the wavelength of the laser in vacuum, respectively. From the apparent diffusion coefficient *D*_app_, the hydrodynamic radius *R*_h_ can be obtained using the Stokes–Einstein relationship $R_{h}=k_{B}T/6\pi \eta_{0}D_{app}$, where *k*_B_ is the Boltzmann constant, *T* is the temperature, and *η*_0_ is the viscosity of the solvent [33,34].

*Electrophoretic Light Scattering (ELS).* Zeta potential (*ζ*_P_) measurements were conducted with a Zetasizer Nano-ZS (Malvern Panalytical Ltd., Malvern, United Kingdom) equipped with a He-Ne laser (*λ* = 633 nm) and an avalanche photodiode detector. The Henry equation in the Smoluchowski approximation was used for zeta potential calculation [35]. *ζ*_P_ values were determined using the Smoluchowski equation $\zeta_{P}=4\pi \eta \upsilon /\varepsilon$, where *η* is the solvent viscosity, *υ* the electrophoretic mobility, and *ε* the dielectric constant of the solvent, and are reported as averages of fifty repeated measurements at a 13° scattering angle and room temperature.

*UV-vis spectroscopy.* UV-vis absorption spectra of the complexes and neat porphyrin and copolymer solutions were recorded between 200 and 800 nm wavelength using a UV-vis NIR double-beam spectrophotometer (Lambda 19 by Perkin Elmer, Waltham, MA, USA) at room temperature. Appropriate dilutions of the samples were performed so as not to exceed maximum acceptable absorbance values. Specifically, the complex solutions were diluted with a factor 1:20 and the neat TPPS 1:100 (final concentration 5.3 μg/mL).

*Fluorescence spectroscopy.* Steady-state fluorescence spectra of the neat and complexed TPPS porphyrin were recorded with a double-grating excitation and a single-grating emission spectrofluorometer (Fluorolog-3, model FL3-21, Jobin Yvon-Spex, Horiba Ltd., Kyoto, Japan) at either room temperature or higher temperatures regulated by a circulating water bath. Excitation wavelength used was *λ* = 515 nm and emission spectra were recorded in the region 535–800 nm, with an increment of 1 nm, using an integration time of 0.1 s, and slit openings of 2 nm for both the excitation and the emitted beam. Under the employed experimental conditions, fluorescence from the TPPS was observed and utilized to extract information regarding its structure, while the neat copolymer solutions did not show any significant fluorescence.

## 3. Results and Discussion

### 3.1. PDMAEMA-b-POEGMA and TPPS Complexation

**Figure 1:**
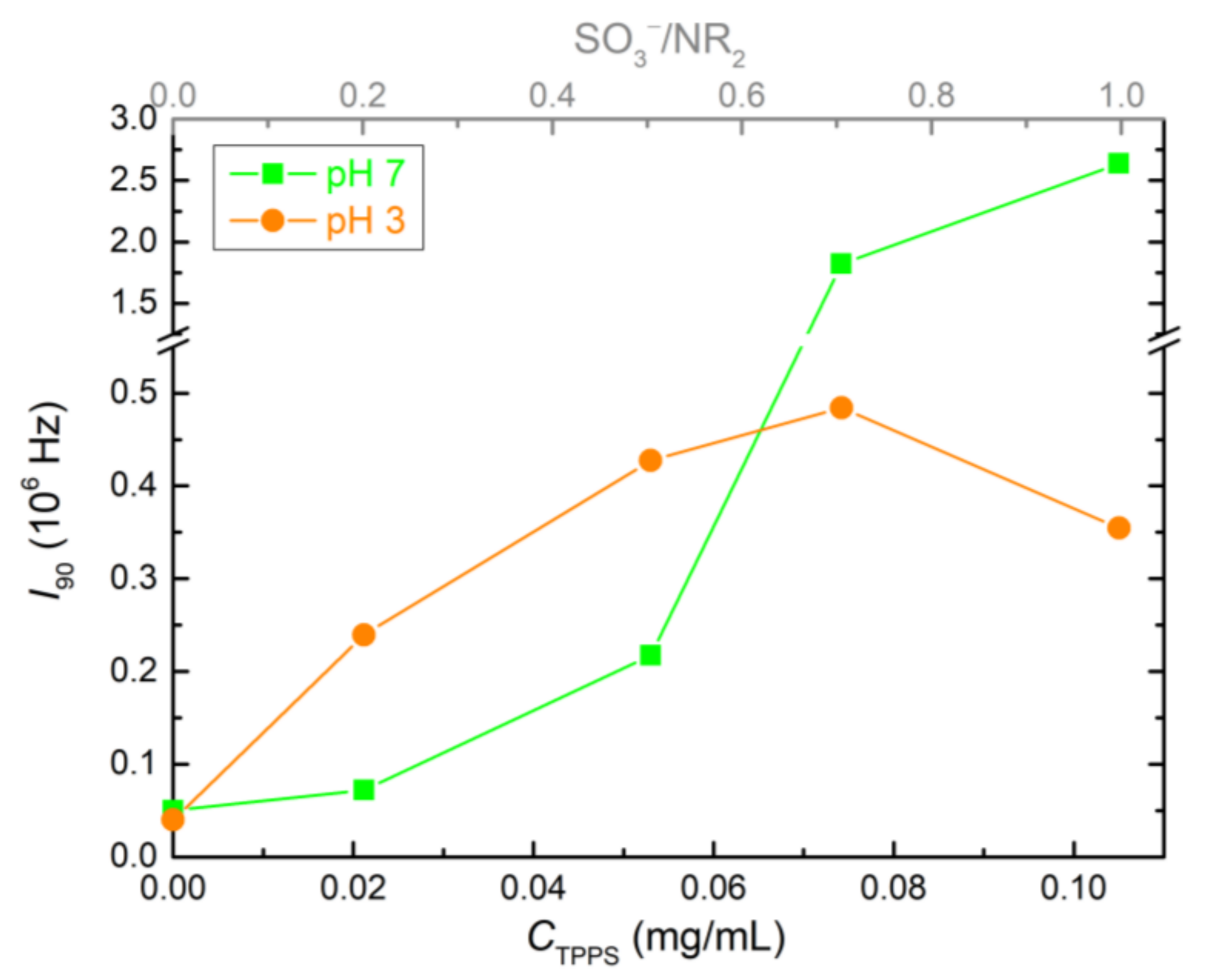
DLS intensity values at 90° *I*_90_ for the DHBC/TPPS complex solutions at pH 7 and 3, as a function of porphyrin concentration *C*_TPPS_ or charged groups ratio SO_3_^−^/NR_2_ (R = CH_3_).

At first, we investigated the formation of PICs through the electrostatic complexation between the PDMAEMA-*b*-POEGMA DHBC and TPPS porphyrin at both pH 7 and 3. The pH value plays a significant role in the protonation state of both the tertiary amine groups of the DHBC, which become fully protonated at pH 3, thus increasing charge density, as well as the pyrrolic nitrogen atoms of the porphyrin molecule that are protonated at pH below 5, thus transforming the porphyrin from its free-base form (H_2_TPPS^4−^) to its diacid form (H_4_TPPS^2−^) [15,16,17]. The solution properties of the stable complex solutions were initially examined by means of DLS measurements, and Figure 1 shows the obtained scattering intensity values derived from measurements at 90° (*I*_90_) as a function of the porphyrin concentration (*C*_TPPS_) or equivalently charged groups ratio (SO_3_^−^/NR_2_, R = CH_3_) at both pH values. Note that the *I*_90_ values at *C*_TPPS_ = 0 correspond to those of the neat DHBC solutions at the same concentration as in the complexes (*C*_DHBC_ = 0.2 mg/mL).

The observed gradual increase in *I*_90_ values at both pH conditions serves as a proof of complexation and PIC formation, since the measured intensity is proportional to the mass of the scattering species in the solution. The apparent decrease at highest *C*_TPPS_ and pH 3 is most probably an undesired effect of the filtration of the samples (a standard practice for DLS measurements so as to remove any large dust particles or aggregates), which is always associated with some degree of solute retention and of course is more pronounced at higher concentrations. At low *C*_TPPS_ values (<0.06 mg/mL), the mass of the complexes formed at pH 3 is larger than the corresponding one at pH 7, a fact that indicates a higher degree of interaction and can be attributed to the increased charge density of the DHBC due to amine protonation. Nevertheless, as the concentration of the porphyrin increases (*C*_TPPS_ > 0.07 mg/mL), an abrupt increase in the mass of the formed PICs at pH 7 is observed and likely denotes some degree of aggregation.

**Figure 2:**
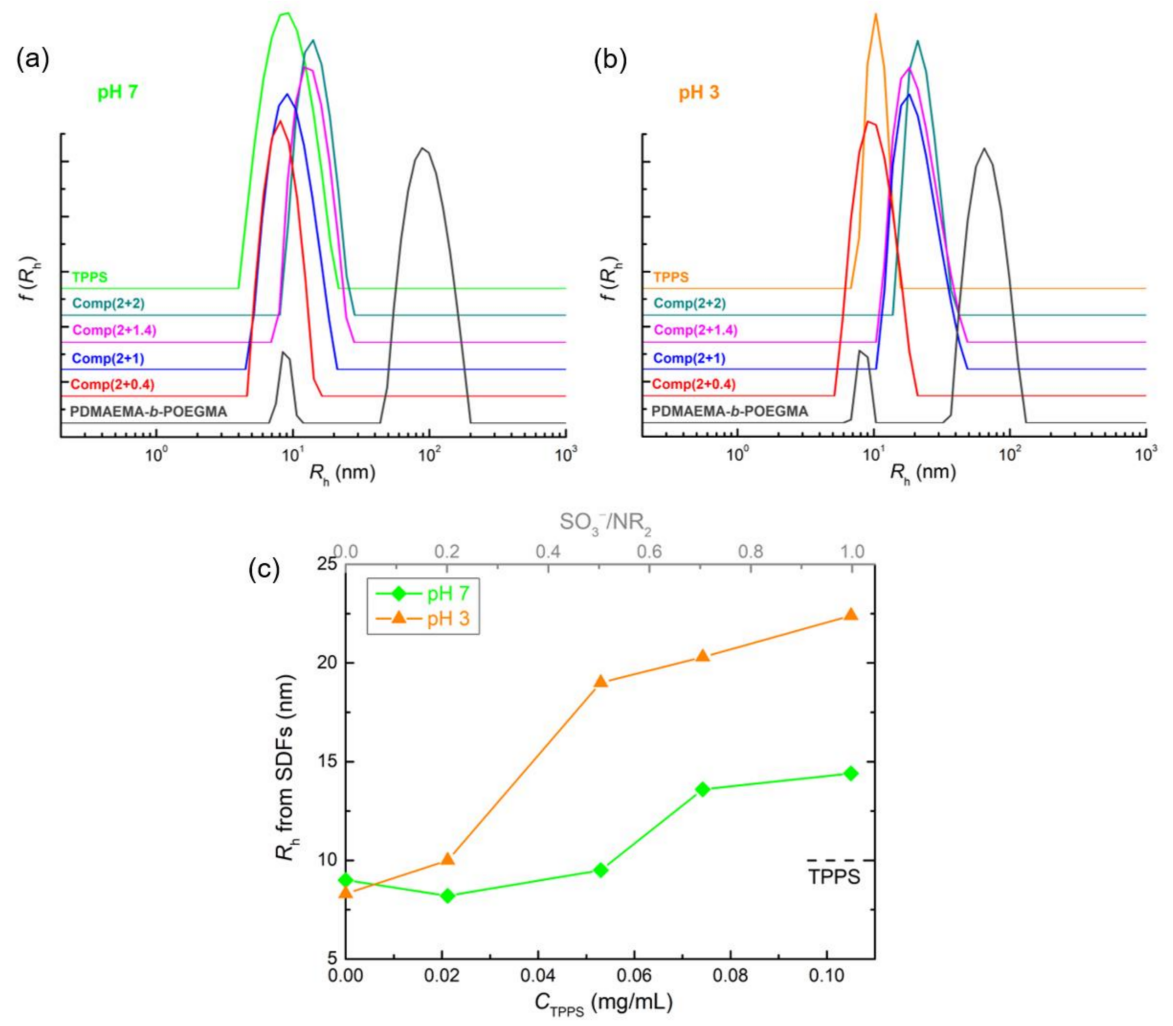
Size distribution functions (SDFs) derived from DLS measurements at 90° for the DHBC/TPPS complex solutions at pH (**a**) 7 and (**b**) 3, along with (**c**) the *R*_h_ values of the individual peaks as a function of *C*_TPPS_ or SO_3_^−^/NR_2_ ratio. The dashed line denotes the corresponding *R*_h_ value of neat TPPS.

Further insight regarding the properties of the complexes can be obtained from the equivalent size distribution functions (SDFs) extracted through the CONTIN regularization of the DLS measurements at 90° that are presented in Figure 2, along with the hydrodynamic radius *R*_h_ values of the individual peaks as a function of *C*_TPPS_ or the SO_3_^−^/NR_2_ ratio at both pH values. The corresponding SDFs of the neat DHBC and TPPS solutions at relevant concentrations (*C*_DHBC_ = 0.2 mg/mL and *C*_TPPS_ = 0.053 mg/mL) are also included for comparison, with the extracted *R*_h_ values being noted in Figure 2c as the point at *C*_TPPS_ = 0 for the DHBC or the dashed line for TPPS.

With respect to the sizes of the species in solution, first of all we should point out that the DHBC exhibits two peaks, one small with an *R*_h_ about 9 nm, which possibly represents the molecularly dissolved copolymer single chains, and a second larger one about 90 or 70 nm depending on the pH that most likely indicates some degree of self-assembly and the formation of multichain aggregates. Although both blocks of the PDMAEMA-*b*-POEGMA copolymer are deemed hydrophilic, hydrophobic interactions stemming from the hydrophobic nature of the polymeric backbone can never be excluded and thus lead to the proposed formation of multichain domains, as also previously observed in analogue systems [36,37]. However, the solutions of the complexes exhibit only one peak in all cases, suggesting the presence of monodisperse PICs with *R*_h_ values ranging from 10 to 20 nm. As it seems upon interaction with the porphyrin the multichain domains of the DHBC breakup—likely because the electrostatic interactions in the system are stronger—and this way, only one type of complexes comprising of TPPS and single copolymer chains are formed. As the concentration of the porphyrin increases, so does the size of the PICs at both pH conditions, indicating further incorporation of TPPS. A more pronounced increase is evident at pH 3, leading to larger complexes, and is probably associated with the higher degree of interaction caused by protonation of the DHBC amine groups. Remarkably, the observed abrupt increase of the PICs mass at pH 7 and high *C*_TPPS_ is not accompanied by a similar increase in size; therefore, it must be correlated with some type of conformational change that leads to more compact structures. One final observation here is that the neat porphyrin has a hydrodynamic radius of about 10 nm, surely significantly larger than its actual molecular size, which is about 20 Å [38]. Hence, we conclude that porphyrin is also aggregated to some extent.

**Figure 3:**
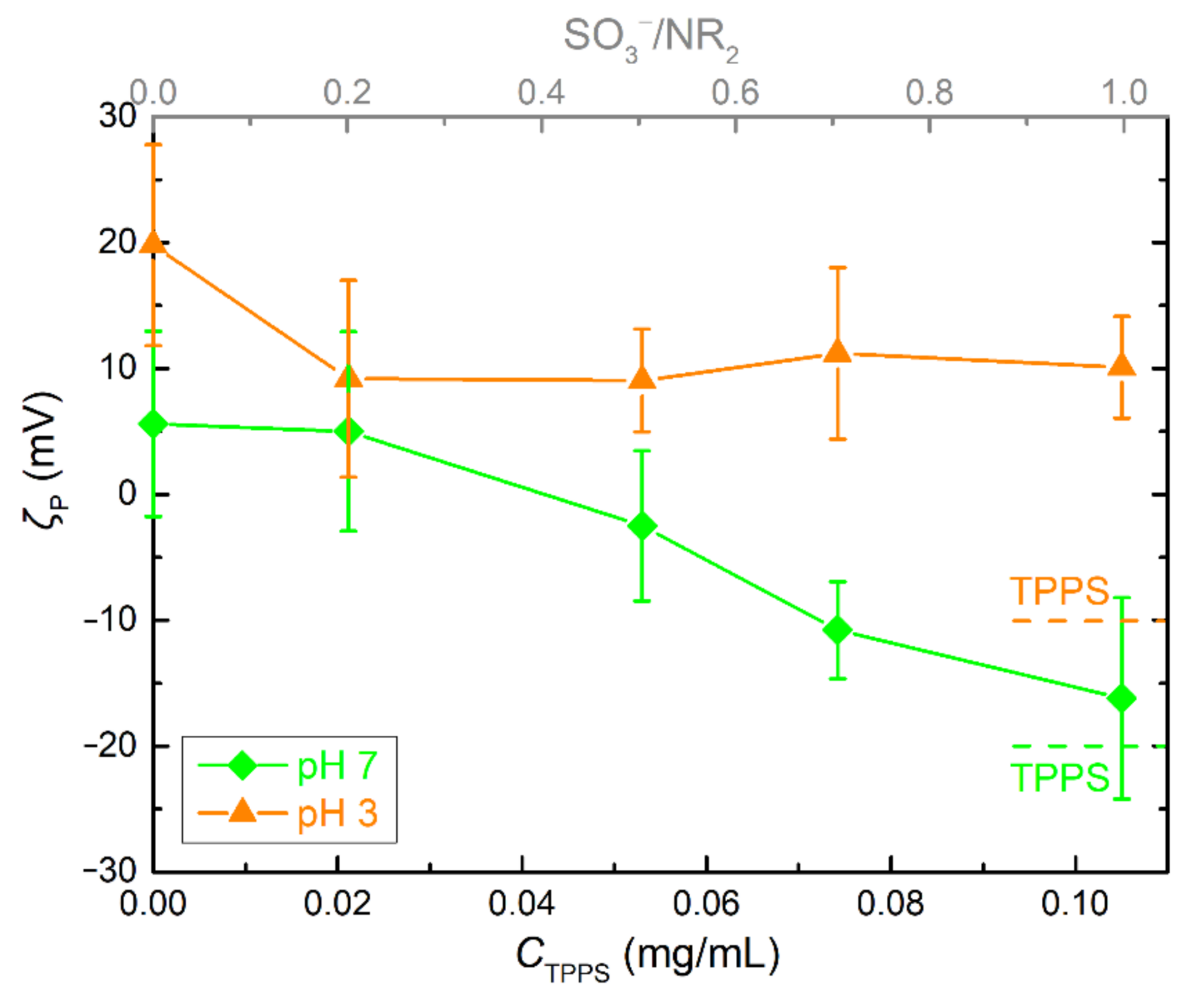
Zeta potential *ζ*_P_ values for the DHBC/TPPS complex solutions at pH 7 and 3, as a function of *C*_TPPS_ or SO_3_^−^/NR_2_ ratio. The dashed lines denote the corresponding *ζ*_P_ values of neat TPPS.

Another crucial characteristic of the formed PICs is their effective charge, which was acquired via electrophoretic light scattering (ELS) measurement, yielding the zeta potential *ζ*_P_ values presented in an identical manner to the previous DLS results in Figure 3. At both pH values, a decrease in *ζ*_P_ values of the complexes in regard to that of the neat DHBC (points at *C*_TPPS_ = 0) is seen, suggesting the neutralization of charges that takes place upon interaction of the two oppositely charged species. At pH 7, we observe a broader change that eventually leads to charge inversion from positive to negative values, or in other words the negative charge of TPPS (*ζ*_P_ ≈ −20 mV) prevails. On the contrary, at pH 3, though the initial decrease is more abrupt, the effective charge of the PICs remains positive for all complex solutions. This is apparently a consequence of the increased charge density of the positive DHBC (as also evident from the corresponding *ζ*_P_ values) and the parallel reduction of TPPS negative charges (note that at pH 3 TPPS has a *ζ*_P_ ≈ −10 mV) due to N protonation. Overall, the number of positive charges in the system is greater at pH 3, thus resulting in positively charged PICs.

**Figure 4:**
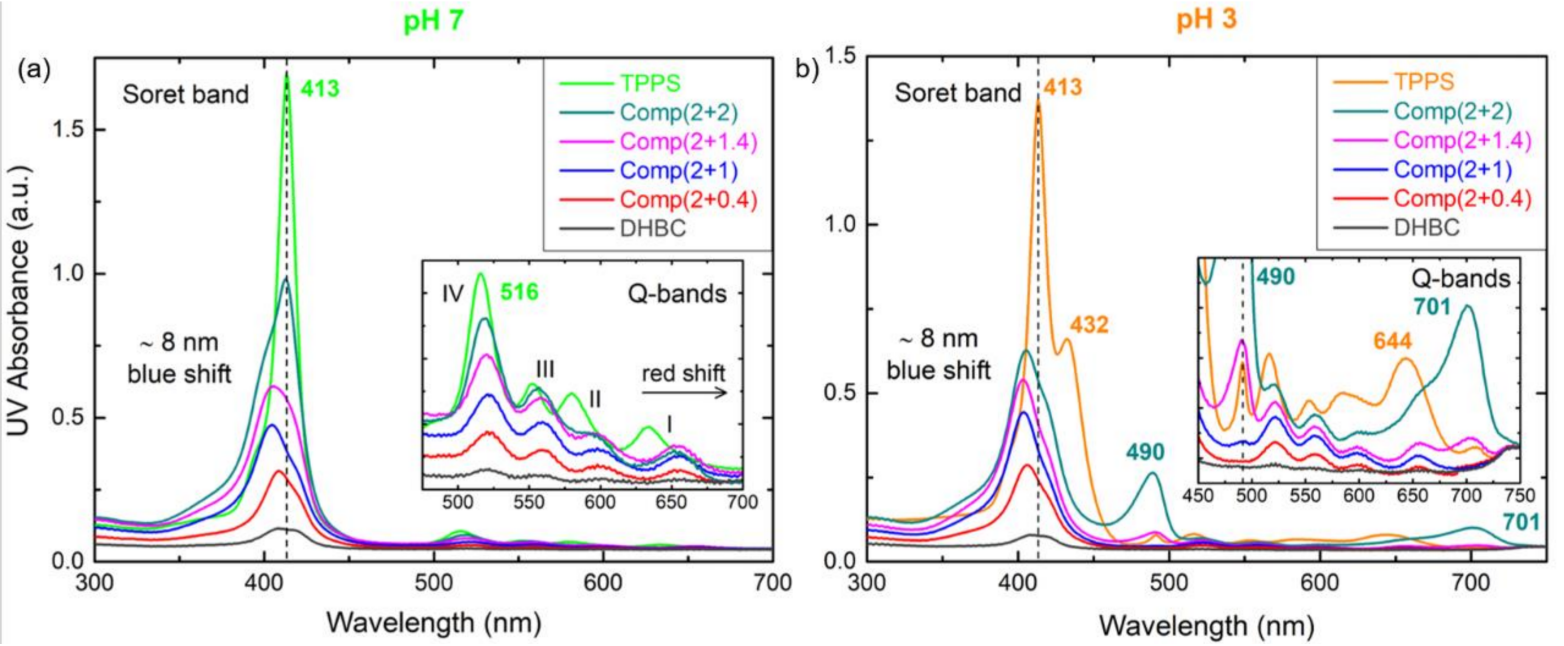
UV-vis spectra of the DHBC/TPPS complex solutions at pH (**a**) 7 and (**b**) 3. The insets show a scaling up of the Q-bands area. The corresponding spectra of neat TPPS and DHBC are also included for comparison.

Of equal importance to their solution characteristics are the optical properties of the formed PICs. For this reason, the complex solutions of the DHBC/TPPS system at both pH conditions were studied by UV-vis spectroscopy and the collected spectra are shown in Figure 4, where the corresponding spectra of the neat DHBC and TPPS solutions are also included for comparison. As expected, the DHBC does not exhibit any significant absorbance, so the observed peaks are attributed to the presence of the porphyrin. The spectroscopic features of TPPS are well known and can be directly correlated with the protonation and aggregation state of the porphyrin molecule [15,16,17,27,29,31,39]. As previously mentioned, at physiological pH values, TPPS exists in its free-base monomeric state (H_2_TPPS^4−^), which at high concentration or increased packing conditions can form face-to-face H-aggregates through *π*–*π* stacking of the molecules. On the other hand, at low pH values, the protonated diacid monomer (H_4_TPPS^2−^) is dominant and these zwitterionic molecules can interact electrostatically via their positive central and negative peripheral charges, forming side-by-side J-aggregates. Both types of TPPS monomers, as well as their aggregated counterparts, have distinctive absorption signatures.

In this case, TPPS solution at pH 7 shows a Soret band located at 413 nm accompanied by a Q-IV band at 516 nm, both indicative of the H_2_TPPS^4−^ monomeric free-base form. In comparison, a gradual blue shift of about 8 nm is observed for the Soret band of the complex solutions, peaking at around 405 nm, while at the same time the Q-bands are slightly shifted to higher wavelengths (red shift). These two findings are related to the formation of H-type aggregates [15,16,17], an apparent consequence of the complexation with the DHBC, which increases the proximity of the porphyrin molecules and thus enhances their aggregation. Moreover, it is worth noting that for the complex solution with the highest *C*_TPPS_, the Soret band shows also significant contribution from the 413 nm peak. This probably signifies the coexistence of H-aggregates and free-base monomers, meaning that some uncomplexed porphyrin molecules are still present in the solution. Therefore, it is possible that the maximum binding capacity of the DHBC has been exceeded.

At pH 3, the neat porphyrin shows a Soret band again peaking at 413 nm—the spectroscopic signature of the free-base monomer—but also a shoulder at 432 nm is observed. This shoulder, along with the distinctive Q-I peak at 644 nm, denote the presence of the diacid monomer [15,16,17]. Interestingly, it seems that both types of monomeric forms (H_2_TPPS^4−^ and H_4_TPPS^2−^) are simultaneously present in the solution. A possible explanation for this is that the aggregation of the porphyrin molecules hinders their protonation to a full extent. It should be mentioned here that although no signs of H-aggregates can be seen in the spectra of neat TPPS at pH 7, these measurements were performed on highly diluted solutions (1:100) so as not to exceed maximum allowed absorbance values. Surely, the presence of aggregates in the initial stock solutions (*C*_TPPS_ = 0.53 mg/mL) that were used for the preparation of the PICs cannot be excluded, as was also evidenced by the corresponding hydrodynamic radii (*R*_h_ ≈ 10 nm). In regard to the complexes, the Soret band is once more shifted at 405 nm and the Q-bands are slightly red-shifted, denoting the existence of H-aggregates. Nevertheless, as porphyrin concentration increases the representative peaks of J-aggregates at 490 and 701 nm appear [15,16,17], as clearly obvious in the case of Comp (2 + 2) but also noticeable for the solutions of Comp (2 + 1.4) and neat TPPS, as seen in the inset of Figure 4b. Apparently, at higher *C*_TPPS_ values the number of TPPS molecules participating in the complexes and hence their proximity increases drastically, leading to J-type aggregation. Lastly, no signs of uncomplexed TPPS can be discerned in this pH value, one more indication of the increased interaction capability of the DHBC due to the protonation of its amine groups.

**Figure 5:**
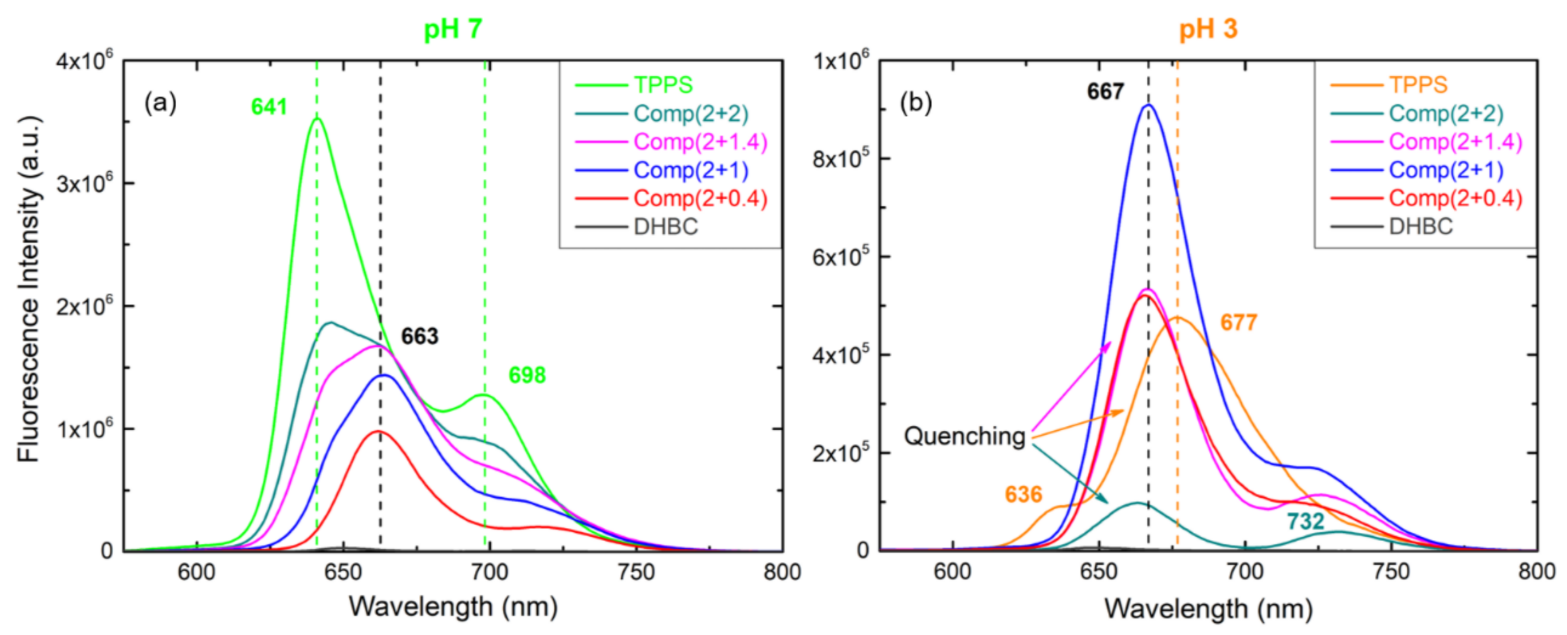
Fluorescence spectra of the DHBC/TPPS complex solutions at pH (**a**) 7 and (**b**) 3. The corresponding spectra of neat TPPS and DHBC are also included for comparison.

Fluorescence spectroscopy measurements of the same PIC solutions were also performed and the relative emission spectra are shown in Figure 5. Overall, the fluorescence spectral features corroborate the findings from the UV-vis measurements. At pH 7, the neat TPPS solution displays a double peak at 641 and 698 nm, which is typical of the free-base monomer [16,27,29]. A red shift of about 20 nm is observed for the complex solutions with the main emission peak appearing at 663 nm, thus signifying the presence of H-aggregates. Notably, as porphyrin concentration increases, contribution from the 641 nm peak becomes more and more prominent, culminating in the case of the Comp (2 + 2) solution, again indicating uncomplexed TPPS monomeric molecules. Respectively, at pH 3 a significant decrease in the fluorescence intensity of the neat TPPS is observed and the main peak is located around 677 nm, both spectral traits of the diacid H_4_TPPS^2−^ form [16,27,29]. However, a small shoulder at 636 nm probably correlated with the free-base monomer is still discerned. The obtained emission spectra for the solutions of the PICs resemble the ones at pH 7, with the main peak about 667 nm (existence of H-aggregates). For the two complex solutions with higher porphyrin concentration, extended quenching is observed along with the appearance of the peak at 732 nm, transitions that can be attributed to the formation of J-aggregates [16,27,29].

### 3.2. QPDMAEMA-b-POEGMA and TPPS complexation

**Figure 6:**
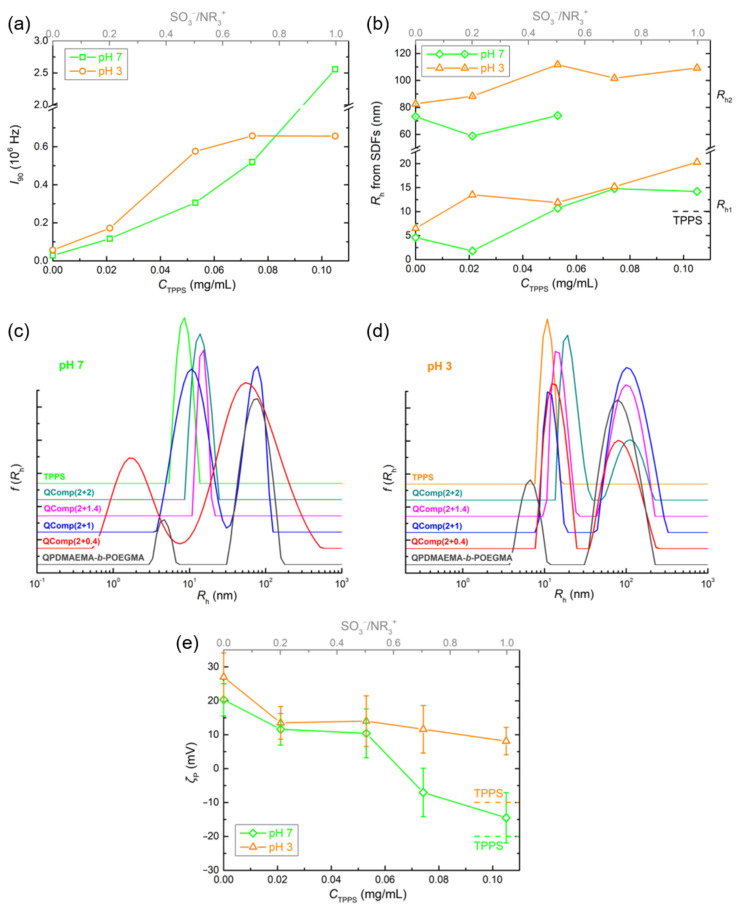
Collective DLS and ELS results in regard to (**a**) the scattering intensity at 90°, (**b**) the hydrodynamic radius derived from (**c**,**d**) the corresponding size distribution functions (SDFs) at 90°, and (**e**) zeta potential values for the QDHBC/TPPS complex solutions at pH 7 and 3, as a function of porphyrin concentration *C*_TPPS_ or charged groups ratio SO_3_^−^/NR_3_^+^ (R = CH_3_).

Undoubtedly, electrostatic interactions play the most crucial role during the coupling of (macro)molecular species bearing opposite charges and essentially govern not only the complexation process itself but the properties of the resulting nanoparticles as well. Ergo, any change in the charged state of the individual components is expected to influence the final outcome and for this reason is worth investigating. On these grounds, we also studied the interaction of the quaternized homologue QPDMAEMA-*b*-POEGMA copolymer, which is a strong polyelectrolyte independently of pH with the TPPS porphyrin at both acidic and neutral pH conditions. Figure 6 presents the collective DLS and ELS results attained for the QDHBC/TPPS system in regard to scattering intensity, hydrodynamic radius, size distribution and effective charge, as a function of *C*_TPPS_ or equivalently the SO_3_^−^/NR_3_^+^ (R = CH_3_) charged groups ratio at both pH 7 and 3.

As seen in Figure 6a, the mass of the complexes (proportional to the scattering intensity) increases with increasing *C*_TPPS_, validating once again their formation. Somewhat larger *I*_90_ values are observed at pH 3 than at 7, although in this case the charge density of the QDHBC is the same at both pH values. Possibly, the higher mass of the complexes at pH 3 could be attributed to the differences in the protonation and/or aggregation state of the porphyrin molecule, as well as hydration state of the copolymer due to the additional CH_3_ group. Moreover, generally higher scattering values are recorded in comparison to the previous DHBC/TPPS system (see Figure S1), indicating an analogous difference in the mass of the corresponding PICs. Quite interestingly, abrupt increase in *I*_90_ at pH 7 occurs only at the highest porphyrin concentration, demonstrating a more gradual and less intense transition to more compact conformations.

A better understanding of the observed changes in the mass of the complexes can be gained by examining the corresponding differences in their size and size distributions (Figure 6b–d). First of all, the QDHBC shows two peaks signifying the presence of single copolymer chains with a size of about 5 nm, along with multichain domains/aggregates giving peaks about 70 or 80 nm (at pH 7 or 3, respectively), as was the case for the precursor copolymer. The remarkable difference for this system is that the majority of the complex solutions also exhibit two peaks, which most likely denote the presence of two different types of complexes in regard to the morphology of the QDHBC. In other words, both the single chains and the multichain aggregates of the copolymer form complexes with the porphyrin. At pH 7 and high *C*_TPPS_, the second peak is no longer discerned, suggesting that the multichain aggregates break up as the interaction with the TPPS becomes more prominent and only one type of PIC is preserved. On the contrary, at pH 3 both types of PICs coexist throughout the whole range of mixing ratios (i.e., component analogies), a fact that clearly justifies the observed increased mass. As it seems the different protonation/aggregation state of TPPS at pH 3 entails a different type of interaction with the quaternized copolymer, presumably weaker, that apparently is not able to cause the dissociation of the multichain domains. A small increase in most peaks (apart from *R*_h2_ at pH 7) can be seen, possibly indicating the incorporation of more porphyrin molecules.

As far as the effective charge of the complexes is concerned, the *ζ*_P_ values decrease as *C*_TPPS_ increases (Figure 6e), a direct and anticipated consequence of the charge neutralization that takes place upon interaction. Again, the overall change is greater at pH 7 than at 3 owing to the increased number of positive charges related to TPPS protonation and aggregation state.

**Figure 7:**
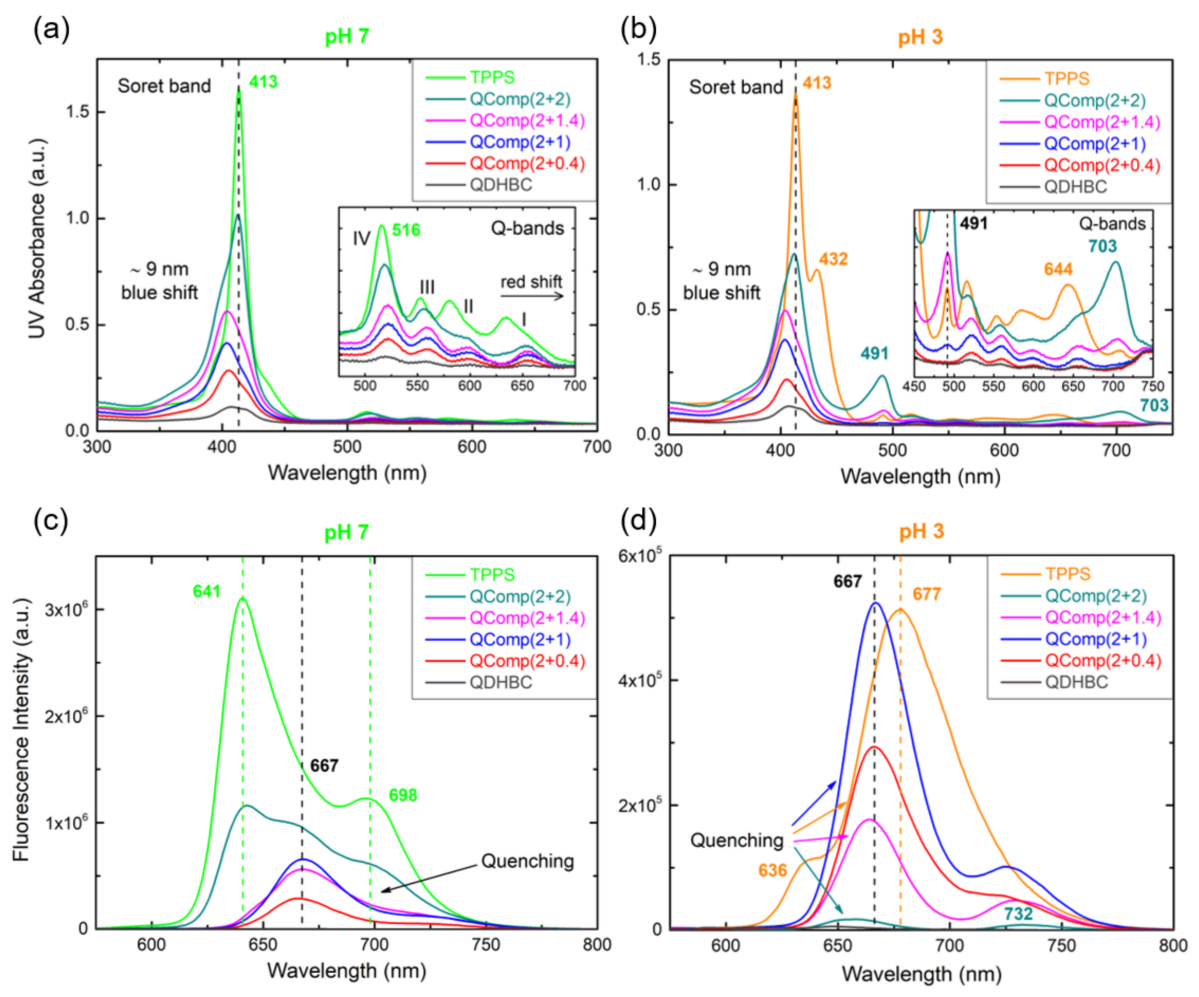
UV-vis (**a**,**b**) and fluorescence (**c**,**d**) spectra of the QDHBC/TPPS complex solutions at pH 7 (left) and 3 (right). The insets in the top row show a scaling up of the Q-bands area. The corresponding spectra of neat TPPS and QDHBC are also included for comparison.

Additional information about the state of the porphyrin molecules in the complexes can be derived from the UV-vis and fluorescence spectra of the QDHBC/TPPS system at both pH values shown in Figure 7. Absorbance spectra at pH 7 are almost identical to the ones measured for the previous system, characterized by a blue-shifted Soret band peaking about 404 nm and somewhat red-shifted Q-bands indicative of H-aggregation. Similarly, the same observations regarding the formation of H-type aggregates can be made at pH 3, while at high porphyrin concentrations the distinctive spectral features of J-aggregates are ascertained (peaks at 491 and 703 nm). Note that at both pH conditions and highest porphyrin content, the contribution of the 413 nm peak is clearly evident in the Soret band of the complexes, revealing the existence of uncomplexed TPPS and thus implying that the maximum binding capacity of the quaternized copolymer has been reached. Fluorescence spectroscopy measurements confirm the attained rationalization about H-aggregation by showing at both pH 7 and 3 a main peak located around 667 nm. Nevertheless, a more pronounced quenching of the emission signal occurs in this case, meaning that the aggregation of the porphyrin molecules is more extensive. Furthermore, at pH 3 and high *C*_TPPS_, telltale signs of J-aggregates (extensive quenching and peak at 732 nm) can be seen.

### 3.3. Comparison of the Two Systems in Different Conditions

**Scheme 2:**
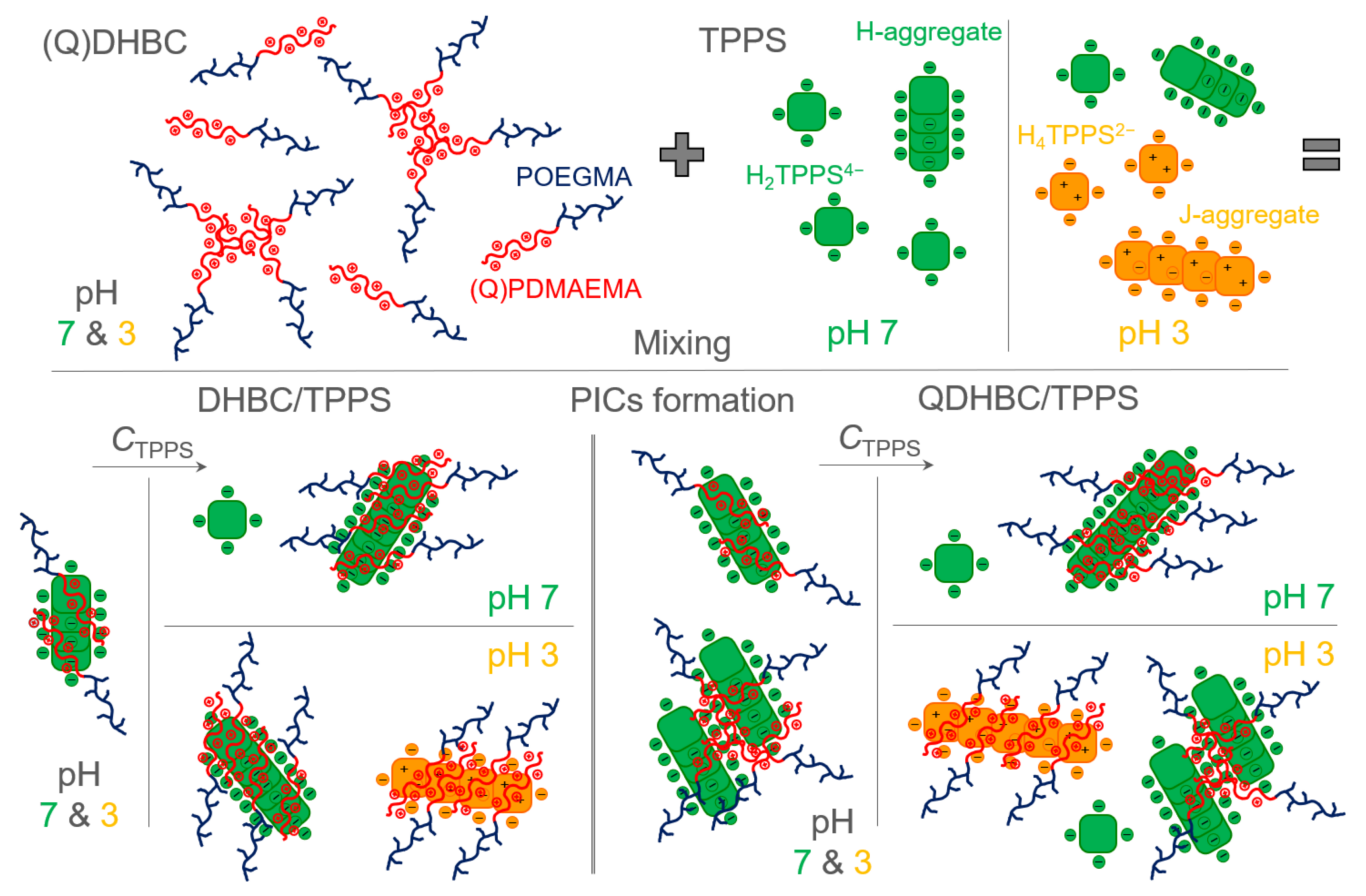
Proposed interaction illustration for the formation of PICs between the DHBC or its quaternized counterpart QDHBC and TPPS porphyrin at different pH values and porphyrin contents.

Certainly, the sum of the results presented thus far emphasizes the importance of self-assembly processes governed by electrostatic and/or hydrophobic interactions when one deals with such nanostructured hybrid systems. According to our observations, the nature of the macromolecular copolymer in regard to its protonation degree or equivalent charge density, as well as solvation state and latent hydrophobic nature that leads to the formation of multichain domains, along with the complementary features of the porphyrin molecule mainly involving its protonation and aggregation state, constitute the key factors influencing the structure and properties of the resulting PICs. Recapitulating, both the precursor PDMAEMA-*b*-POEGMA copolymer and its quaternized counterpart form multichain aggregates at either pH 7 or 3 (apparently stemming from hydrophobic backbone interactions) coexisting with single polymeric chains in solution. In an analogue manner, TPPS porphyrin exhibits two protonation states, that is, the free-base (H_2_TPPS^4−^) at pH 7 or diacid (H_4_TPPS^2−^) at pH 3 monomer, which in turn self-assemble when in close proximity into H- or J-type structures due to *π*–*π* stacking or electrostatic binding, respectively. Upon mixing of these different species in the case of the DHBC/TPPS system at low porphyrin concentration and both pH conditions, monodisperse PICs comprising H-aggregated TPPS molecules and singe DHBC chains are formed. As the porphyrin content increases, so does its aggregation, leading to more compact complexed H-aggregates at pH 7, integrating more of TPPS molecules and eventually exceeding DHBC binding capacity. Accordingly, at pH 3 both H- and J-aggregates are incorporated in the resulting complexes, since both types are present in the corresponding neat TPPS solution, while the increased charge density of the DHBC causes a higher degree of interaction (no uncomplexed TPPS is discerned). When it comes to the quaternized DHBC, it seems that the consecutive change in the hydration state of the copolymer is associated with more stable multichain aggregates that do not easily dissociate during complexation. This way, two types of distinct PICs are formed at both pH values and low *C*_TPPS_ corresponding to single and multichain copolymer–porphyrin complexes. However, as the porphyrin concentration becomes higher at pH 7 only the single-chain type of PICs remains incorporating more TPPS molecules (suggesting that porphyrin aggregation is more prominent), while at pH 3 both types coincide and even complexes of J-aggregated TPPS are observed. Still, at both pH conditions spectroscopic signs of uncomplexed porphyrin are recorded, a fact that implies a reduced binding affinity for the quaternized copolymer in comparison to the precursor DHBC, a counterintuitive finding that points out the significance of the intrinsic polymeric solution properties. This proposed interaction scenario is schematically depicted in the following illustration (Scheme 2).

### 3.4. Temperature Effect on the Formed PICs

**Figure 8:**
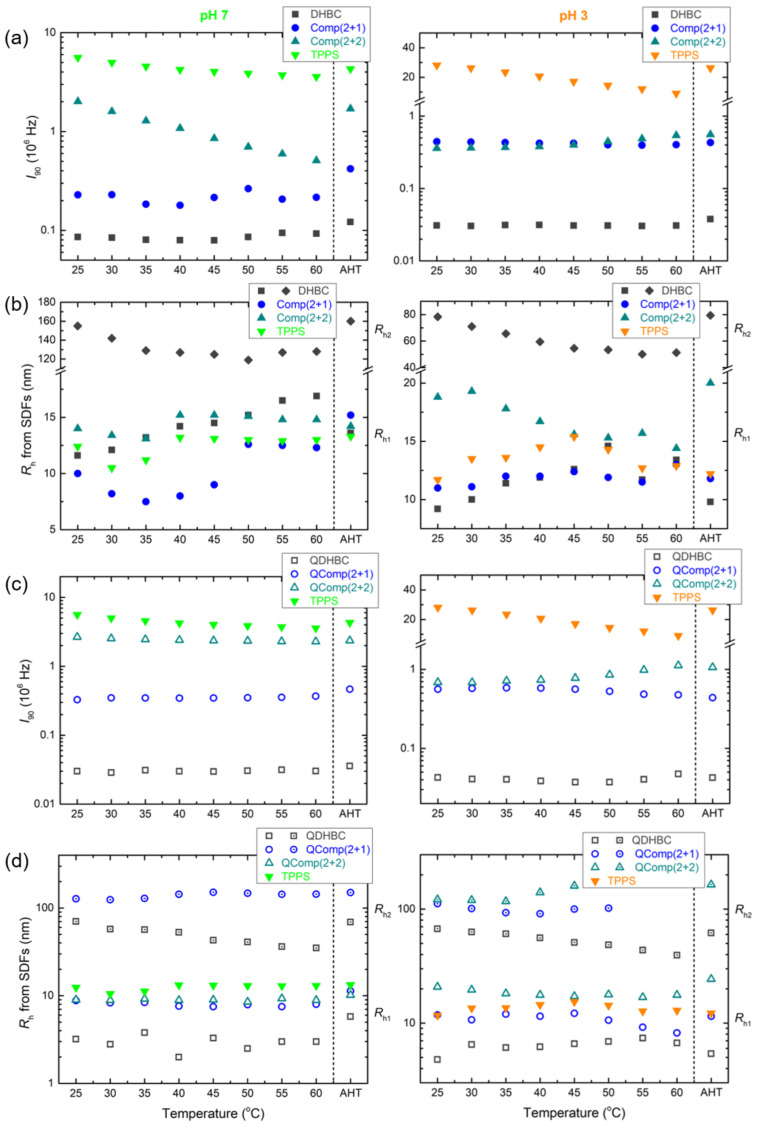
DLS results in regard to (**a**,**c**) the scattering intensity at 90° and (**b**,**d**) the hydrodynamic radius derived from the corresponding size distribution functions (SDFs) at 90°, for the complex solutions (Q)Comp (2 + 1) and (Q)Comp (2 + 2) of the (Q)DHBC/TPPS systems at pH 7 (left) and 3 (right) at different temperatures ranging from 25 to 60 ℃ (5 ℃ step), and cooled back at 25 ℃ after heat treatment (AHT). The corresponding values of neat (Q)DHBC and TPPS are also included for comparison.

**Figure 9:**
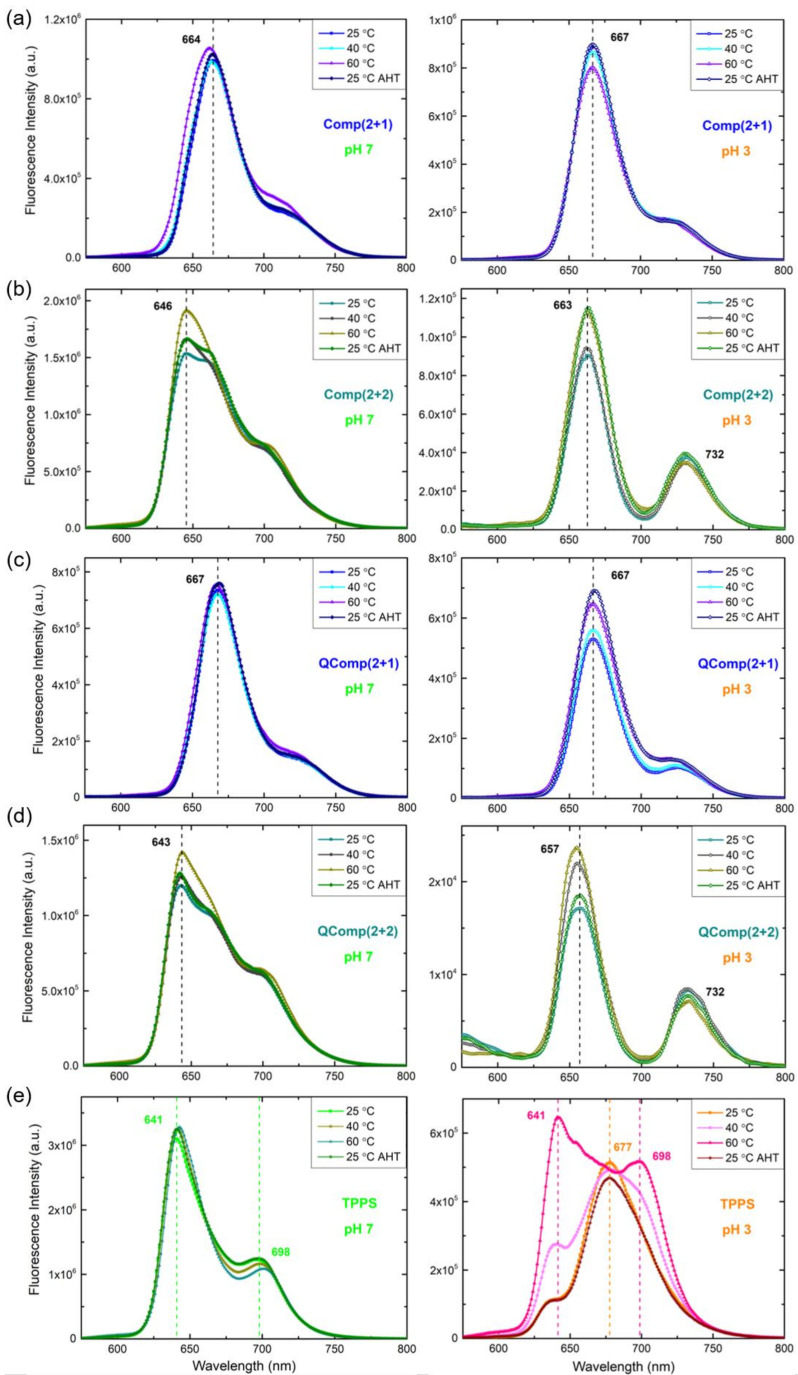
Fluorescence spectra of the complex solutions (**a**,**c**) (Q)Comp (2 + 1) and (**b**,**d**) (Q)Comp (2 + 2) of the (Q)DHBC/TPPS systems at pH 7 (left) and 3 (right) at different temperatures: 25, 40, 60 ℃, and 25 ℃ after heat treatment (AHT). (**e**) The corresponding spectra of neat TPPS for comparison.

The ability of photosensitizers intended for clinical applications to respond to externally applied stimuli such as temperature is of particular interest, since it can open new treatment pathways and even enable combination of therapeutic protocols or methods, thus enhancing their efficiency potential [4,40]. To this end, we investigated the effect of temperature on the already formed PICs of both systems under study. Figure 8 shows the summary of DLS results regarding the values of the scattering intensity *I*_90_ and the hydrodynamic radius *R*_h_ derived from the corresponding size distribution functions (SDFs)-presented in Figures S2–S4 obtained via measurements performed at 90° and different temperatures ranging from 25 to 60 ℃ (with a 5 ℃ increment), for two representative solutions of complexes-Comp (2 + 1) and Comp (2 + 2)-of the DHBC or QDHBC/TPPS system at both pH 7 and 3. The corresponding values of the neat copolymers and porphyrin solutions are also included for comparison. In a similar manner, fluorescence spectra of the same representative solutions measured at temperatures of 25, 40, 60 ℃, and back at 25 ℃ after heat treatment are displayed in Figure 9, along with the corresponding spectra of neat TPPS.

At first, the effect of temperature on the pure constituents of the complex systems is presented. For both block copolymers, their mass (proportional to *I*_90_) seems rather constant throughout the range of studied temperatures. In regard to their size, although the *R*_h_ of the first peak (single copolymer chains) shows small changes, an apparent decrease in the *R*_h_ of the second peak is observed (see also Figure S4). This fact indicates that the multichain aggregates shrink as temperature increases, most probably because hydrophobic interactions become stronger. When temperature drops back to ambient conditions, all sizes are restored. As far as porphyrin is concerned, its size is almost unaffected but a systematic decrease of its mass can be discerned, especially in the case of pH 3, where it is more pronounced. This decrease of mass is also accompanied by a significant transition in the respective fluorescence spectra, with the one at 60 ℃ showing clear signs of the free-base monomer (Figure 9e). Apparently, disaggregation (at least to some extent) of the porphyrin H-aggregates-present at both pH values, as previously discussed-takes place, which is eventually reestablished when the solution cools down.

For the complex solutions of the DHBC/TPPS system, only small changes in their mass are observed, apart from the noticeable decrease of the scattering intensity in the case of the Comp (2 + 2) solution at pH 7, which in combination with the parallel increase in emission intensity could denote a dissociation of the aggregated porphyrin molecules. At the same time, their sizes are either stable or suggest slight shrinking as a consequence of enhanced hydrophobic interactions. This seems to also be the case for the smaller population of complexes of the QDHBC/TPPS system, the one that corresponds to single copolymer chain–porphyrin complexes. Remarkably, the larger complexes that are correlated with the copolymer multichain aggregates either diminish or even disappear completely upon heating. This change is also expressed as an increase in the fluorescence intensity or in other words dequenching, denoting possible disaggregation of TPPS. Most likely at higher temperatures, the interaction between the copolymer and porphyrin molecules is more favorable, facilitated also by the apparent dissociation of porphyrin aggregates, thus leading to the dissociation of the multichain complexes. Overall, the increase in temperature showed that the formed PICs are susceptible to conformational rearrangements so as to compensate for the loss of hydration due to the stronger hydrophobic interactions.

## 4. Conclusions

The electrostatic complexation between the DHBC PDMAEMA-*b*-POEGMA and the corresponding quaternized strong block polyelectrolyte QPDMAEMA-*b*-POEGMA with TPPS porphyrin led to the formation of nanostructured photoactive PICs. Both the solution and optical properties and the morphology and structure of these complexed hybrid species proved to be dependent on the protonation and aggregation state of their original constituents, the pH of the solution, and the analogy between the two components. Monodisperse small PICs were formed in the case of the DHBC/TPPS system at both pH 7 and 3, consisting of single copolymer chains and H- or J-type aggregates of TPPS depending on the pH. The increase in TPPS content resulted in more compact complexed porphyrin aggregates. Respectively, the analogous QDHBC/TPPS system was characterized by the presence of two different types of complexes, those resulting from the interaction of the single chains or the multichain aggregates of the quaternized copolymer and the corresponding H-aggregates of TPPS at both pH values and low porphyrin contents. At higher TPPS concentration, increased aggregation of the porphyrin occurred, subsequently affecting the structure of the complexes and thus leading to larger H- or even J-type complexed aggregates. The optical properties of the PICs for both systems were directly correlated with the intrinsic protonation and/or aggregation state of TPPS, which was evidently further enhanced upon interaction with the copolymers. Actually, the quaternized counterpart caused a greater degree of porphyrin aggregation compared to the precursor DHBC. Finally, the formed PICs responded to the increase in temperature by appropriate conformational rearrangements that led to disaggregation of the complexed porphyrin in the case of multichain–porphyrin complexes or more compact structures (shrinking) for the single-chain ones.

## Figures and Tables

**Table 1:** Molecular characteristics of PDMAEMA-*b*-POEGMA and QPDMAEMA-*b*-POEGMA copolymers.

Copolymer	*M*_w_ (10^4^ g/mol) ^a^	*M*_w_/*M*_n_ ^a^	(Q)PDMAEMA Content (wt%)	POEGMA Content (wt%)	(Q)PDMAEMA Monomeric Units	POEGMA Monomeric Units
PDMAEMA-*b*-POEGMA	2.29	1.34	33 ^b^	67 ^b^	48 ^c^	32 ^c^
QPDMAEMA-*b*-POEGMA	2.97		48 ^d^	52 ^d^		

^a^ Determined by SEC; ^b^ determined by ^1^H NMR; ^c^ calculated based on the *M*_n_ of the corresponding monomeric units; ^d^ calculated assuming 100% quaternization and the composition of the precursor copolymer.

**Table 2:** Characteristics of the complex solutions of the DHBC or the QDHBC and TPPS porphyrin.

Sample Name	*C*_(Q)DHBC_ (mg/mL)	*C*_TPPS_ (mg/mL)	SO_3_^−^/NR_2_ or NR_3_^+ a^
(Q)Comp(2 + 0.4)	0.2	0.021	20%
(Q)Comp(2 + 1)		0.053	50%
(Q)Comp(2 + 1.4)		0.074	70%
(Q)Comp(2 + 2)		0.105	100%

^a^ R = CH_3._

## Data Availability

Data presented in this study are available on reasonable request from the corresponding author.

## Supplementary Materials

The following supporting information can be downloaded at: https://www.mdpi.com/article/10.3390/polym14235186/s1, Figure S1: Comparison of DLS scattering intensity values at 90° *I*_90_ for the DHBC/TPPS (closed symbols) and the QDHBC/TPPS (open symbols) complex solutions at pH 7 and 3, as a function of porphyrin concentration *C*_TPPS_ or charged groups ratio SO_3_^−^/NR_2_ or NR_3_^+^ (R = CH_3_).; Figure S2: Size distribution functions (SDFs) derived from DLS measurement at 90° for the complex solutions (a, b) Comp (2 + 1) and (c, d) Comp (2 + 2) of the DHBC/TPPS system at pH 7 (left) and 3 (right) at different temperatures ranging from 25 to 60 ℃ (5 ℃ step), and cooled back to 25 ℃ after heat treatment (AHT); Figure S3: Size distribution functions (SDFs) derived from DLS measurement at 90° for the complex solutions (a,b) QComp (2 + 1) and (c, d) QComp (2 + 2) of the QDHBC/TPPS system at pH 7 (left) and 3 (right) at different temperatures ranging from 25 to 60 ℃ (5 ℃ step), and cooled back to 25 ℃ after heat treatment (AHT); Figure S4: Size distribution functions (SDFs) derived from DLS measurement at 90° for the neat (a,b) DHBC, (c,d) QDHBC and (e,f) TPPS solutions at pH 7 (left) and 3 (right) at different temperatures ranging from 25 to 60 ℃ (5 ℃ step), and cooled back to 25 ℃ after heat treatment (AHT).
