# Supplementary material for: Development of Double Hydrophilic Block Copolymer/Porphyrin Polyion Complex Micelles towards Photofunctional Nanoparticles
Source: Polymers (Basel). 2022 Nov 29;14(23):5186. doi: 10.3390/polym14235186 (PMC9735875; doi:10.3390/polym14235186)
Supplement: Supplementary file 1 [file polymers-14-05186-s001.zip › polymers-2034663-supplementary.pdf]

Supporting Information for:

Development of Double Hydrophilic Block Copolymer/Porphyrin Polyion Complex Micelles Towards Photofunctional Nanoparticles

Maria Karayianni, Dimitra Koufi and Stergios Pispas *

Theoretical and Physical Chemistry Institute, National Hellenic Research Foundation, 48 Vassileos Constantinou Avenue, 116 35 Athens, Greece

* Correspondence: pispas@eie.gr

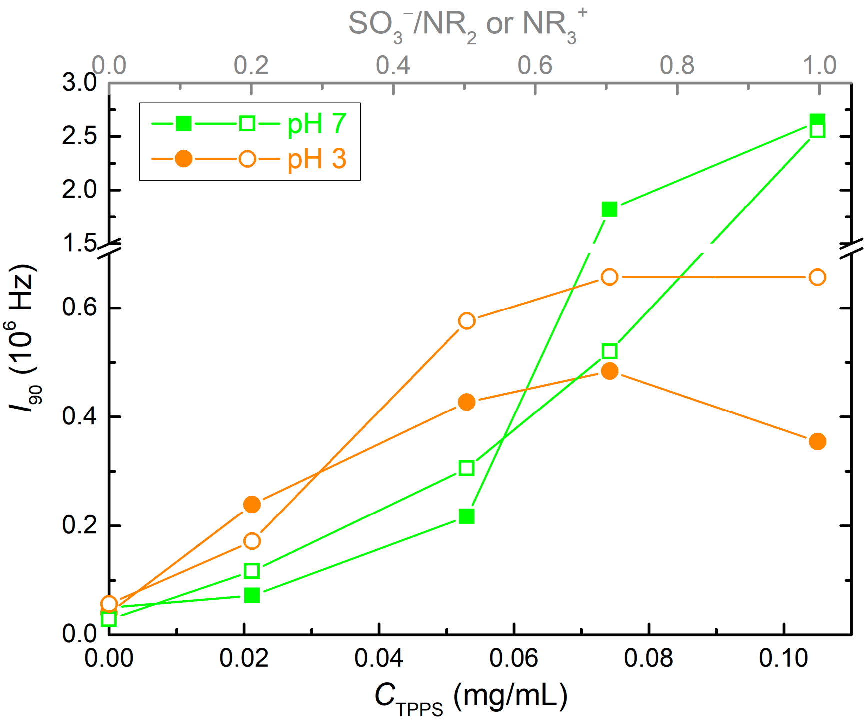

Figure S1. Comparison of DLS scattering intensity values at 90° I_{90} for the DHBC/TPPS (closed symbols) and the QDHBC/TPPS (open symbols) complex solutions at pH 7 and 3, as a function of porphyrin concentration C_{TPPS} or charged groups ratio SO_3^-/NR_2 or NR_3^+ (R = CH_3).

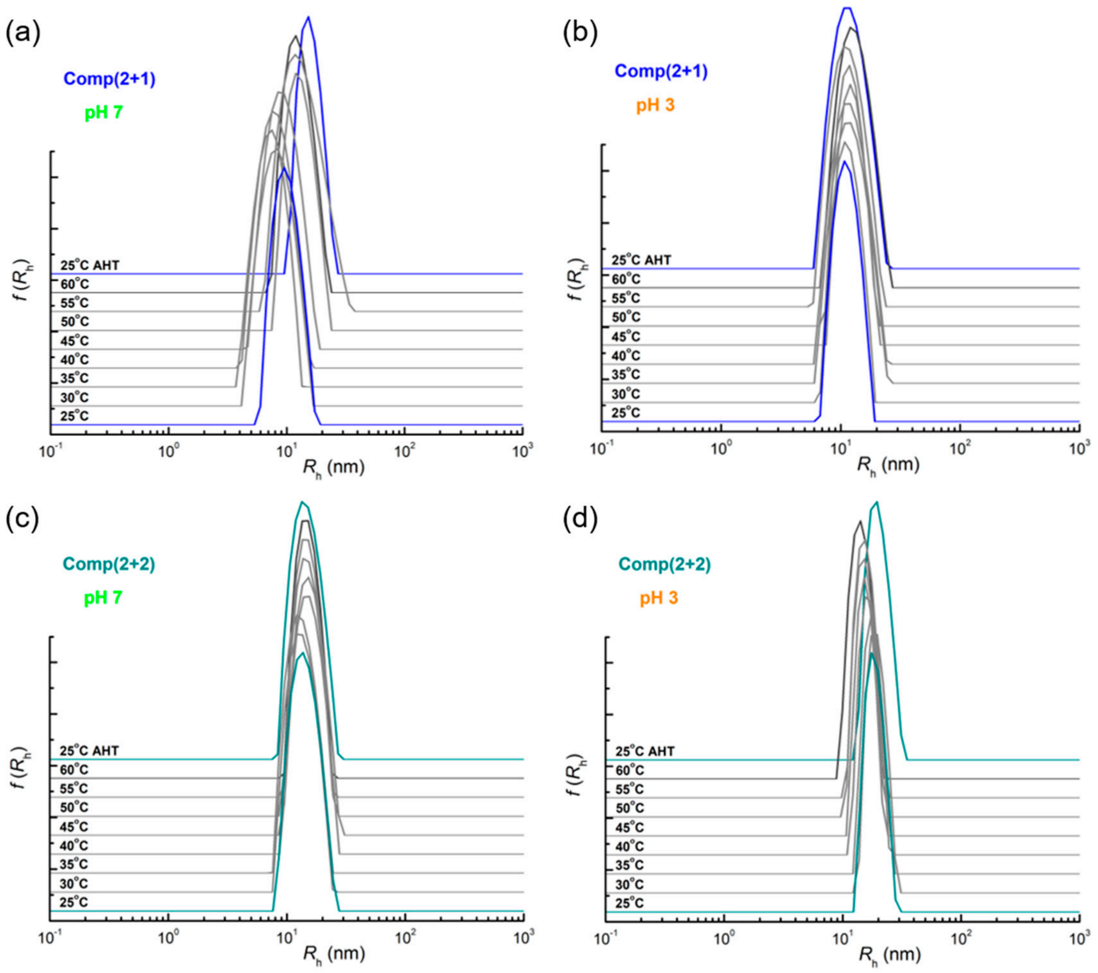

Figure S2. Size distribution functions (SDFs) derived from DLS measurement at 90° for the complex solutions (a, b) Comp(2+1) and (c, d) Comp(2+2) of the DHBC/TPPS system at pH 7 (left) and 3 (right) at different temperatures ranging from 25 to 60 °C (5 °C step), and cooled back to 25 °C after heat treatment (AHT).

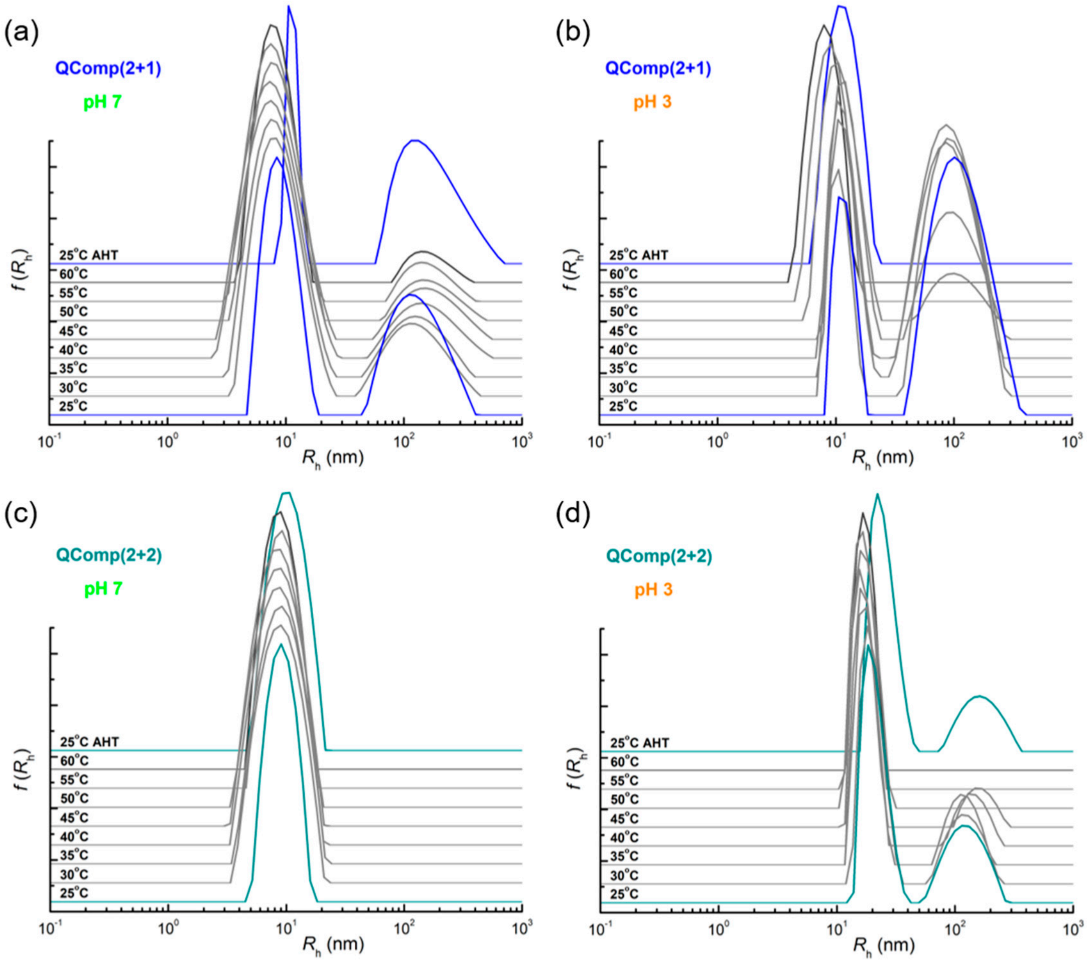

Figure S3. Size distribution functions (SDFs) derived from DLS measurement at 90° for the complex solutions (a, b) QComp(2+1) and (c, d) QComp(2+2) of the QDHBC/TPPS system at pH 7 (left) and 3 (right) at different temperatures ranging from 25 to 60 °C (5 °C step), and cooled back to 25 °C after heat treatment (AHT).

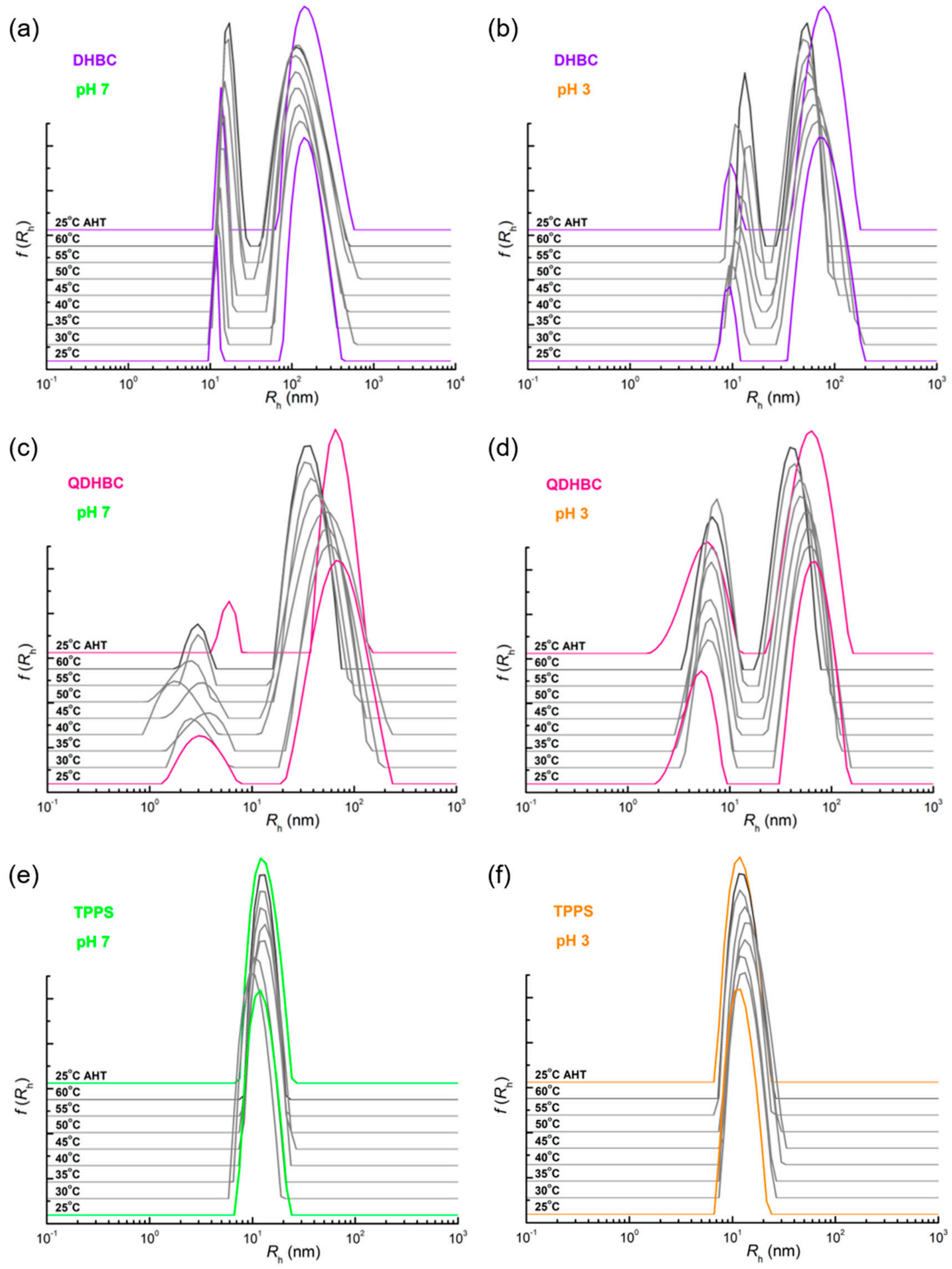

Figure S4. Size distribution functions (SDFs) derived from DLS measurement at 90° for the neat (a, b) DHBC, (c, d) QDHBC and (e, f) TPPS solutions at pH 7 (left) and 3 (right) at different temperatures ranging from 25 to 60 °C (5 °C step), and cooled back to 25 °C after heat treatment (AHT).
